# Hepatic Stellate Cell–Specific METTL3 Deficiency Promotes Hepatocellular Carcinoma Progression via BMP10–SMAD1/5/8 Signaling

**DOI:** 10.1158/2767-9764.CRC-25-0761

**Published:** 2026-05-13

**Authors:** Shanshan Yu, Yiwang Zhang, Yanli Li, Lijie Pan, Xiaoqi Liang, Wanru Meng, Shuai Dong, Yuetong Sun, Xiao Liu, Linsen Ye, Qi Zhang, Yan Xu

**Affiliations:** 1 Biotherapy Centre, The Third Affiliated Hospital, Sun Yat-sen University, Guangzhou, China.; 2 Department of Infectious Diseases, The Third Affiliated Hospital, Sun Yat-sen University, Guangzhou, China.; 3 Department of Pathology, The Third Affiliated Hospital, Sun Yat-sen University, Guangzhou, China.; 4 Department of Laboratory Medicine, The Sixth Affiliated Hospital, School of Medicine, South China University of Technology, Foshan, China.; 5 The Laboratory Animal Center, Sun Yat-sen University, Guangzhou, China.; 6 Cell-Gene Therapy Translational Medicine Research Centre, The Third Affiliated Hospital, Sun Yat-sen University, Guangzhou, China.; 7 Department of Anesthesiology, The Third Affiliated Hospital, Sun Yat-sen University, Guangzhou, China.; 8 Department of Hepatic Surgery and Liver Transplantation Center, The Third Affiliated Hospital, Sun Yat-sen University, Guangzhou, China.; 9 Guangdong Provincial Key Laboratory of Liver Disease Research, The Third Affiliated Hospital, Sun Yat-sen University, Guangzhou, China.

## Abstract

Hepatic stellate cells (HSCs) and their derived cancer-associated fibroblasts (CAFs) are integral to the development, progression, and prognosis of hepatocellular carcinoma (HCC). However, the mechanisms underlying the transformation of HSCs into CAFs and their subsequent roles in HCC remain incompletely understood. The expression of METTL3 was analyzed in fibroblasts infiltrating into human HCC specimens compared with adjacent nontumor tissues. The effects of HSC-specific METTL3 deficiency on HCC were analyzed by orthotopically implanting hepatoma cells into the fibrotic mouse livers, as well as through direct and indirect co-culture systems. We found that the expression of METTL3, the methyltransferase responsible for RNA N6-methyladenosine (m^6^A) modification, was downregulated in fibroblasts infiltrating into HCC compared with that of adjacent nontumor tissues. Orthotopical implantation of HCC cells in the fibrotic liver showed that HSC-specific METTL3 deficiency significantly increased tumor burden. Furthermore, conditioned medium treatment and mixed coculture confirmed that METTL3 deletion in HSCs promoted HCC proliferation and migration *in vitro*, demonstrating that METTL3 deficiency in HSCs accelerated HCC progression in the fibrotic liver. Mechanistically, METTL3 deficiency reduced m^6^A modification and expression of bone morphogenetic protein 10 (BMP10), a known tumor suppressor. Similar to the effects of METTL3 deficiency, BMP10 knockdown also promoted hepatoma cell growth, whereas recombinant BMP10 (rBMP10) inhibited it. Notably, BMP10 overexpression or rBMP10 supplementation effectively mitigated the tumor-promoting effects of METTL3-deficient HSCs. BMP10’s effects were mediated through SMAD1/5/8 phosphorylation. Collectively, our study reveals a novel crosstalk between HSCs and hepatoma cells via the METTL3/m^6^A–BMP10–SMAD1/5/8 axis, highlighting a potential therapeutic target for HCC.

**Significance:**

In this study, we found that HSC-specific METTL3 deficiency significantly accelerated HCC progression in the fibrotic liver. Mechanistically, METTL3 deficiency reduced m^6^A modification and expression of BMP10 and the downstream SMAD1/5/8 phosphorylation. This study reveals a novel crosstalk between HSCs and hepatoma cells, suggesting a potential therapeutic target for HCC.

## Introduction

Hepatocellular carcinoma (HCC) is one of the most common cancers globally, characterized by high mortality and poor prognosis, posing a major challenge to healthcare systems worldwide (1, 2). Though significant therapeutic advances have been made for HCC in recent years, the overall survival rate of HCC is still very low (5-year survival rate is 18% worldwide; ref. 3). One major characteristic of HCC is that 80% to 90% of HCC cases develop on a background of liver fibrosis/cirrhosis (3). The underlying fibrosis/cirrhosis creates a unique tumor microenvironment (TME), including an abundant extracellular matrix (ECM), a diverse range of cancer-associated fibroblasts (CAFs), and a special immune microenvironment. Increasing evidence has revealed that the fibrotic TME contributes to the initiation, progression, and therapeutic response of HCC (4). Among other things, the abundant and highly heterogeneous CAFs secrete multiple inflammatory cytokines and growth factors, creating a tumor-promoting environment that facilitates tumor growth, metastasis, drug resistance, and recurrence (4–6). Lineage tracing and single-cell transcriptomic analysis have shown that the liver-resident hepatic stellate cells (HSCs) generate ∼85% of all CAFs in liver tumors (7). However, the functional role and underlying mechanisms of CAFs in HCC remain poorly elucidated, and targeted anticancer strategies targeting CAFs are still limited.

N6-methyladenosine (m^6^A) modification, one of the most abundant chemical modifications in eukaryotic RNAs, is dynamically and reversibly regulated by 3 groups of factors under various physiologic and pathophysiologic conditions. Methyltransferases, also called “writers,” including METTL3, METTL14, WTAP, RBM15, ZC3H13, and KIAA1429, are responsible for adding a methyl group to the nitrogen atom at the sixth position of adenosine, thereby increasing the m^6^A methylation level of the associated RNAs. Among these, METTL3 is a core component with catalytic activity. In recent years, we and others have reported that METTL3-mediated m^6^A methylation is involved in various liver diseases, including hepatic ischemia–reperfusion injury (8), liver fibrosis (9, 10), nonalcoholic fatty liver disease (11, 12), and liver cancer (13, 14). Previously, we found that METTL3 deficiency attenuated HSC activation and alleviated liver fibrosis (10). We are wondering whether METTL3/m^6^A in HSCs and their derived CAFs also plays a functional role in the HCC microenvironment.

In the present study, we aimed to investigate the effects of METTL3 knockout (KO) in HSCs on HCC progression. We discovered that METTL3 expression was downregulated in activated HSCs infiltrating human HCC tissues compared with adjacent nontumor tissues. METTL3 deficiency exacerbated tumor formation and growth in an orthotopic mouse model of HCC with underlying liver fibrosis. Additionally, knocking down METTL3 in HSCs accelerated the proliferation and migration of HCC cells *in vitro*. Further mechanistic studies revealed that bone morphogenetic protein 10 (BMP10) was a direct target of m^6^A methylation. Knocking down BMP10 in HSCs promoted the proliferation and migration of HCC cells, whereas recombinant BMP10 (rBMP10) protein had the opposite effect. Ultimately, we demonstrated that BMP10 suppressed the HCC tumorigenesis and progression via the SMAD1/5/8 pathway. Our findings uncovered a METTL3/m^6^A–BMP10–SMAD1/5/8 axis involved in the tumorigenesis and progression of HCC, offering a potential therapeutic strategy for future HCC treatment.

## Materials and Methods

### Human tissue samples

This study was approved by the Ethics Committee of the Third Affiliated Hospital of Sun Yat-sen University, with the ethical approval number RG2024-207-01. All research was conducted in accordance with both the Declaration of Helsinki and Declaration of Istanbul. Written informed consent was obtained from all patients involved in the study. Human HCC samples and surrounding nontumor tissues were collected during surgical resection.

### Animals and orthotopic HCC xenograft model in fibrotic mouse livers

All mice were of the C57BL/6J background and housed in a specific pathogen–free facility with a 12-hour light/dark cycle. Food and water were provided *ad libitum*. The *Mettl3*^*flox/flox*^ mice were generously provided by Professor Qi Zhou (15). *Lrat*-*Cre* mice were purchased from GemPharmatech. *Mettl3*^*flox/flox*^/*Lrat*-*Cre* mice [*Mettl3* conditional KO (cKO)] were generated by crossing *Mettl3*^*flox/flox*^ mice with heterozygous *Lrat*-*Cre* mice. *Lrat*-*Cre*–negative littermates were used as wild-type (WT) controls (control). Animal procedures were approved by the Institutional Animal Care and Use Committee of the Third Affiliated Hospital of Sun Yat-sen University (ethical approval number IACUC-F3-21-0907).

For the orthotopic implantation of the HCC mouse model, mouse hepatoma cells Hepa1-6 (RRID: CVCL_0327, provided by Cell Bank, Chinese Academy of Sciences, Shanghai, China) were directly implanted into the median lobe of control and *Mettl3* cKO mice with liver fibrosis. To induce liver fibrosis, 6- to 8-week-old male mice were gavaged with carbon tetrachloride (CCl_4_, 2 mL/kg of body weight, diluted 2:3 v/v with olive oil) for 4 weeks, 3 times per week. Seventy-two hours after the last CCl_4_ gavage, Hepa1-6 cell transplantation was performed. A total of 1 × 10^6^ Hepa1-6 cells were suspended in 25 μL PBS. Immediately before injection, the cell suspension was mixed with Matrigel (Corning Matrigel, 354230) at a 1:1 (v/v) ratio and kept on ice. The cell/Matrigel suspension was injected into the subcapsular region of the median liver lobe using an insulin injection needle. After needle removal, the injection site surface was covered with Gelfoam for 3 to 5 minutes to minimize bleeding and potential leakage.

### Mouse primary HSC isolation

Mouse primary HSCs were isolated from control and *Mettl3* cKO mice as previously described (10). Briefly, mice were perfused *in situ* via portal vein cannulation with Ethylene Glycol-bis-(2-aminoethylether)-N,N,N′,N′-tetraacetic acid buffer, pronase (Roche, 11459643001), and collagenase (Sigma-Aldrich, C5138). Primary HSCs were purified from cell suspension containing Nycodenz (Accurate Chemical, 1002424) by density gradient centrifugation. The cells were cultured with high-glucose DMEM (Thermo Fisher Scientific, C11995500BT) supplemented with 10% fetal bovine serum (FBS, PAN-Biotech, P30-3302) and 2% penicillin/streptomycin (KeyGEN Biotech, KGY0023) at 37°C in a humidified atmosphere of 5% CO_2_.

### Coculture experiments

Hepatoma cells Hep3B (RRID: CVCL_0326) and Huh7 (RRID: CVCL_0336) were obtained from the Cell Bank, Chinese Academy of Sciences (Shanghai, China), maintained in high-glucose DMEM supplemented with 10% FBS (ExCell Bio, FSP500), and incubated at 37°C in a humidified incubator with 5% CO_2_. Cells were continuously monitored for *Mycoplasma* contamination to ensure that they were free of contamination.

For the coculture experiments, Hep3B or Huh7 cells expressing green fluorescent protein (GFP) were cocultured with LX-2 cells for 48 hours. For proliferation analysis, the number of GFP^+^ hepatoma cells was calculated using a flow cytometer (Beckman, CytoFLEX LX, RRID: SCR_019627).

### Conditioned medium preparation

The human HSC cell line LX-2 (RRID: CVCL_5792) was also provided by Cell Bank (Shanghai, China) and cultured in high-glucose DMEM supplemented with 10% FBS. Cells were continuously monitored for *Mycoplasma* contamination to ensure that they were free of contamination.

To obtain conditioned medium (CM) of HSCs, primary isolated HSCs or LX-2 cells were plated in 10-cm culture dishes at 1 × 10^6^ cells per dish and cultured overnight in high-glucose DMEM containing 10% FBS. Then, the medium was changed to high-glucose DMEM containing 1% FBS. The culture supernatant was collected after 36 hours and filtered through a 0.22-μm membrane before use.

### m^6^A-specific methylated RNA immunoprecipitation with next-generation sequencing

Methylated RNA immunoprecipitation sequencing with next-generation sequencing (MeRIP-seq) was performed as previously described (10). Mouse primary HSCs were isolated and cultured *in vitro* for 7 days for spontaneous activation. Total RNA was extracted, and mRNA was purified. Then, mRNA was fragmented and enriched by m^6^A antibody (Synaptic Systems, 202003, RRID: AB_2279214). Sequencing was conducted by Guangzhou Epibiotek Co., Ltd. Sequencing reads were mapped to the mouse genome (mm10) using HISAT2 2.1.0 (RRID: SCR_015530) (16). Differential m^6^A peaks were identified using the exomePeak R package (17). m^6^A RNA–related genomic features were visualized using Integrative Genomics Viewer (RRID: SCR_011793). Sequencing data are available in the Gene Expression Omnibus database (RRID: SCR_005012) under accession number GSE207908.

### Western blotting

Cell pellets were homogenized in RIPA cell lysis buffer supplemented with protease inhibitor (Roche, 04693132001) and phosphatase inhibitor (Roche, 04906837001). The extracted proteins were separated by 10% SDS-PAGE gel and transferred to nitrocellulose membranes. After incubation with primary antibodies (BMP10, 1:500; p-SMAD 1/5/8, 1:1,000; SMAD1, 1:1,000; GAPDH, 1:1,000) and secondary antibody, the membranes were detected using the ChemiDoc Imaging System (Bio-Rad) using Immobilon ECL Ultra Western HRP Substrate (Millipore, WBULS500). ImageJ software (RRID: SCR_003070) was used to quantify protein expression, and GAPDH served as a loading control. Further information on antibodies used in Western blotting is listed in Supplementary Table S1.

### Quantitative real-time PCR

Total RNA was isolated from cells or liver tissues using TRIzol reagent (Invitrogen, 15596026), and cDNA was synthesized using HiScript III One-Step RT-PCR Kit (Vazyme, R323-01) according to the manufacturer’s instructions. Quantitative real-time PCR (RT-qPCR) was performed in triplicate on LightCycler 480 II (Roche) with ChamQ Universal SYBR qPCR Master Mix (Vazyme, Q711-03). The relative *Gapdh* mRNA level served as an internal control. The primers used for RT-qPCR are listed in Supplementary Table S2.

### m^6^A-RIP-qPCR

m^6^A-RIP-qPCR was performed on LX-2 cells and mouse primary HSCs isolated from control or *Mettl3* cKO mice, as previously described (10) Total RNA was extracted, and poly(A) mRNAs were purified using GenElute mRNA Miniprep Kit (Sigma, MRN70-1KT). mRNA was fragmented and incubated with anti-m^6^A antibody or mouse IgG (as a negative control) for 2 hours at 4°C. Protein A beads and protein G beads were added to the mixture and incubated for another 2 hours at 4°C. RNase inhibitor (Promega, N2611) was added throughout the entire process. mRNA was eluted and extracted via phenol–chloroform extraction. The precipitated mRNAs were reverse-transcribed with HiScript III One-Step RT-PCR Kit (Vazyme, R323-01) according to the manufacturer’s instructions, and m^6^A enrichment was determined via RT-qPCR. Primers used for m^6^A-RIP-qPCR are listed in Supplementary Table S2.

### Statistics

Experimental data were analyzed using GraphPad Prism 8.0.1 (RRID: SCR_002798) and were presented as mean ± SEM. The two-tailed Student *t* test was used for comparisons between two groups, and comparisons between multiple groups were determined using one-way analysis of variance (ANOVA). *P* value < 0.05 was considered to be statistically significant.

Further details on the materials and methods are provided in the Supplementary Materials and Methods and Supplementary Table S3.

## Results

### METTL3 expression was downregulated in CAFs infiltrated into HCC tissues

**Figure 1.**
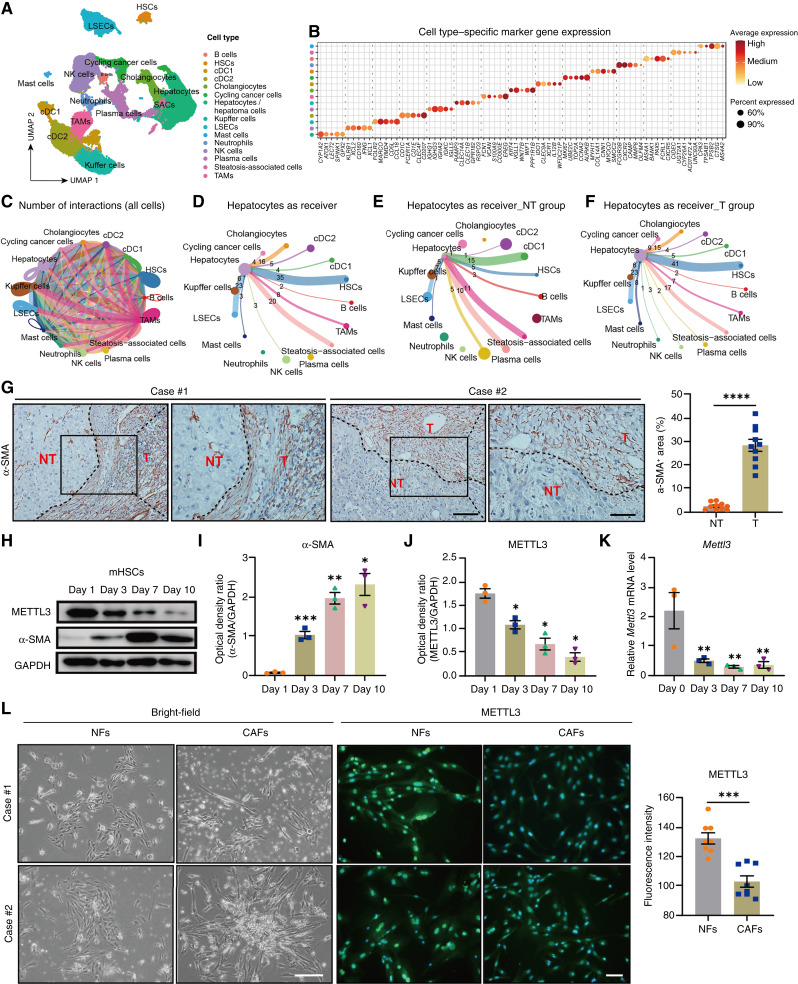
METTL3 expression is downregulated in CAFs infiltrated into HCC tissues. **A,** Uniform Manifold Approximation and Projection for Dimension Reduction (UMAP) with predicted cell annotations depicting cell clusters of the human HCC microenvironment (scRNA-seq data were retrieved from GSE242889). **B,** Dotplot visualizing cell type–specific marker gene expression across different cell populations. **C,** Cell–cell interaction network diagram showing intercellular communication between different cell types in the HCC microenvironment. **D,** Overall cell–cell communication network with hepatocytes as the signal-receiving cell type. **E** and **F,** Cell–cell cross-talk patterns with hepatocytes as recipients in the surrounding nontumor (NT) group (**E**) and the tumor (T) group (**F**). **G,** Representative IHC staining images (left) and quantification (right) of α-SMA in human HCC tissues (T) and nontumor surrounding tissues (NT; scale bars, 50 and 100 μm). **H–K,** Representative Western blots (**H**) with corresponding quantification (**I** and **J**) for α-SMA and METTL3 and RT-qPCR for *Mettl3* (**K**) in mouse primary HSCs activated during *in vitro* culture. GAPDH was used as the loading control for the Western blot, and the *Gapdh* mRNA level was used as the internal control for RT-qPCR (also hereafter in similar experiments). **L,** Representative bright-field images (left) and representative fluorescent images (middle) with corresponding quantification (right) for METTL3 immunofluorescence staining of human primary CAFs isolated from human HCC tumors and fibroblasts isolated from adjacent nontumor tissues (NF; scale bars, 200 μm for bright-field images and 100 μm for fluorescent images). Cell nuclei were counterstained with Hoechst 33342 (also hereafter in similar experiments). Data in **G** and **I**–**L** are presented as mean ± SEM with the indicated significance (*, *P* < 0.05; **, *P* < 0.01; ***, *P* < 0.001; ****, *P* < 0.0001). cDC, conventional dendritic cell; LSEC, liver sinusoidal endothelial cell; mHSCs, mouse primary HSCs; SAC, steatosis-associated cell; TAM, tumor-associated macrophage.

Fibroblasts infiltrating the HCC microenvironment are thought to contribute to tumor initiation and progression. By reanalyzing published single-cell RNA sequencing (scRNA-seq) data (GSE242889), we found that HSCs were indeed most closely associated with hepatoma cells in the HCC microenvironment and hepatoma cells received the strongest signals from HSCs (Fig. 1A–D). Comparing with adjacent nontumor tissues, communication between HSCs and hepatocytes or hepatoma cells was significantly enhanced (Fig. 1E and F). Then, we performed α-SMA (an HSC/CAF activation marker) immunohistochemistry (IHC) staining in human HCC tumors and surrounding nontumor tissues to confirm changes in HSC infiltration in HCC tissues. Consistent with previous reports (18), α-SMA–positive cells were more abundant in HCC tumor tissues compared with adjacent nontumor tissues (Fig. 1G). Though CAFs in HCC are a highly heterogeneous population that can originate from disparate precursors (including portal fibroblasts, HSCs, endothelial cells, vascular smooth muscle cells, and bone marrow–derived mesenchymal stromal cells), previous lineage tracing studies showed that activated HSCs are the primary source of CAFs in HCC (accounting for ∼85% CAFs in HCC; (7). Given that METTL3-mediated m^6^A modification plays a key role in HSC activation during liver fibrosis (9, 10), we wonder about METTL3’s function in CAFs during HCC tumorigenesis and progression.

We first isolated primary HSCs from WT mice and cultured them *in vitro* to allow for spontaneous activation. We found that METTL3 expression gradually decreased during spontaneous activation of HSCs (Fig. 1H–K). Next, we detected the expression pattern of METTL3 in α-SMA–positive CAFs infiltrated in HCC. We isolated human primary HSCs/CAFs from HCC tissues and fibroblasts from paired adjacent nontumor tissues and assessed the expression of METTL3 by immunofluorescence. Similar to the findings in mouse primary HSCs, METTL3 expression in primary CAFs from HCC tissues was significantly lower than that in fibroblasts from adjacent nontumor tissues (Fig. 1L). The above data indicated that activated HSCs were more abundant in HCC tissues and that METTL3 expression was downregulated in activated HSCs infiltrating HCC.

### HSC-specific *Mettl3* KO promoted HCC progression

**Figure 2.**
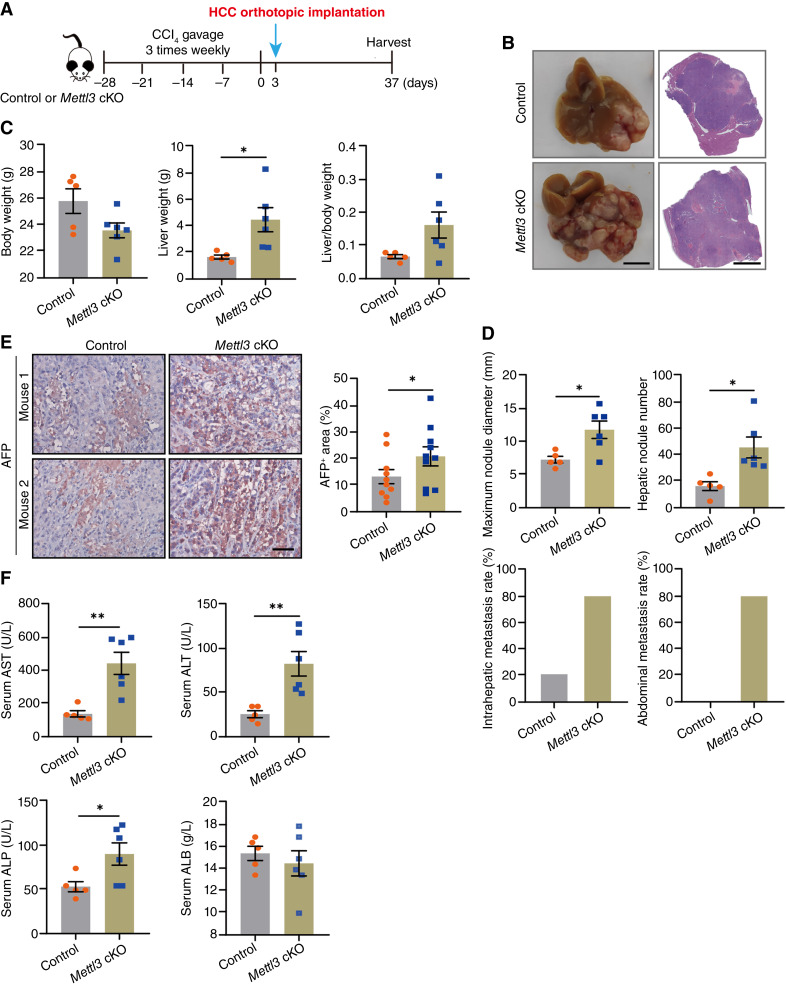
HSC-specific METTL3 KO promotes HCC progression in mice with liver fibrosis. Liver fibrosis of *Mettl3*^*flox/flox*^ (control) or HSC-specific *Mettl3*-KO (*Mettl3* cKO) mice was induced by CCl_4_ gavage for 4 weeks. Three days after the last CCl_4_ gavage, Hepa1-6 cells were transplanted into the liver. Samples were collected 34 days later and analyzed (*n* = 5 for the control group and *n* = 6 for the *Mettl3* cKO group). **A,** Schematic diagram of the experimental design. **B,** Representative whole-liver gross appearance (left) and hematoxylin and eosin staining photographs (right) of indicated groups [scale bars, 1 cm (left) and 3 mm (right)]. **C,** Body weight, liver weight, and liver weight to body weight ratios of mice in the control and *Mettl3* cKO groups. **D,** Maximum nodule diameter, hepatic nodule number, intrahepatic metastasis rate, and abdominal metastasis rate of the control and *Mettl3* cKO groups. **E,** Representative IHC staining images (left) and quantification (right) for AFP (scale bar, 50 μm). **F,** Serum levels of AST, ALT, ALP, and ALB of control and *Mettl3* cKO mice. Data in **C–F** are presented as mean ± SEM with the indicated significance (*, *P* < 0.05; **, *P* < 0.01). AFP, α-fetoprotein; ALB, albumin; ALP, alkaline phosphatase; ALT, alanine aminotransferase; AST, aspartic transaminase.

Next, we used the *Lrat-Cre*–mediated HSC-specific *Mettl3*-KO (*Mettl3* cKO) transgenic mice we created previously (10) to investigate the functional role of METTL3/m^6^A in HSCs during HCC progression. We first induced liver fibrosis in control and *Mettl3* cKO male mice by chronic CCl_4_ treatment. Then, mouse hepatoma cells Hepa1-6 were orthotopically inoculated into the median lobe of the fibrotic liver. The difference in tumor growth and metastasis between control and *Mettl3* cKO mice was analyzed 34 days after orthotopic hepatoma cell implantation (Fig. 2A). The liver gross appearance and histologic examination showed that HSC-specific METTL3 KO dramatically promoted the tumor formation and growth (Fig. 2B), along with lower body weight, higher liver weight, and higher liver weight to body weight ratio (Fig. 2C). In addition, the maximum tumor nodule diameter, intrahepatic nodule number, intrahepatic metastasis rate, and abdominal metastasis rate were all higher in the *Mettl3* cKO group (Fig. 2D), accompanied with more α-fetoprotein–positive hepatoma cells (Fig. 2E) and higher serum levels of aspartate aminotransferase, alanine aminotransferase, and alkaline phosphatase (Fig. 2F). These results demonstrated that HSC-specific METTL3 deficiency accelerated HCC progression in the fibrotic liver.

**Figure 3.**
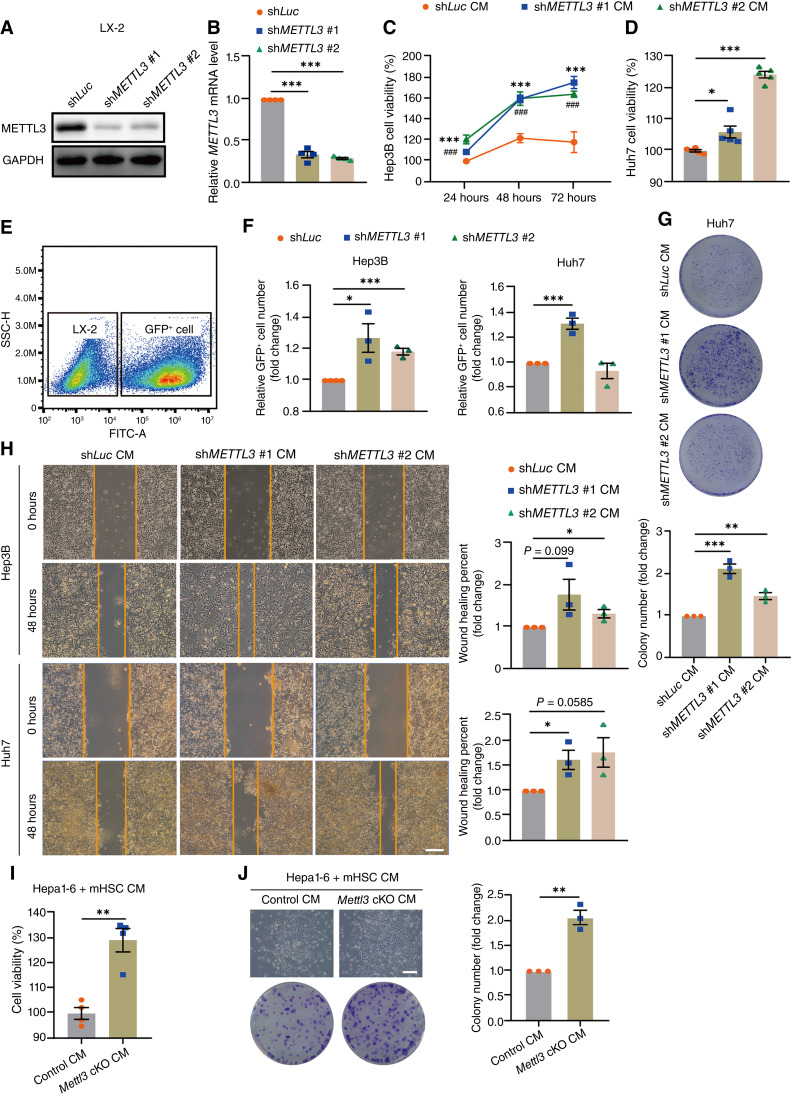
HSC-specific METTL3 deficiency promotes the proliferation of hepatoma cells *in vitro*. **A,** Representative Western blots for METTL3 in LX-2 cells transduced with control [shRNA targeting firefly luciferase (sh*Luc*), also hereafter in similar experiments] or 2 independent shRNAs targeting *METTL3* (sh*METTL3* #1 and sh*METTL3* #2). **B,** RT-qPCR for *METTL3* in control and *METTL3*-knockdown LX-2 cells. **C,** CCK8 assay analyzing the cell proliferation of Hep3B cells cultured within the CM from control and *METTL3*-knockdown LX-2 cells for the indicated time. **D,** CCK8 assay analyzing the cell proliferation of Huh7 cells cultured within CM from indicated LX-2 cells for 48 hours. **E** and **F,** GFP-transduced Hep3B cells or Huh7 cells were directly cocultured with sh*METTL3* LX-2 or *shLuc* LX-2: (**E**) representative flow cytometer plot showing the number and proportion of GFP^−^ LX-2 cells and GFP^+^ hepatoma cells; (**F**) quantification of the number of Hep3B or Huh7 cells cocultured with sh*Luc* or sh*METTL3* LX-2 for 48 hours. Values were normalized to the sh*Luc* group. **G,** Representative images (up) and quantification (bottom) of crystal violet staining for colony-forming assays for Huh7 cells cultured within the indicated LX-2 CM for 7 days. **H,** Representative images (left) and quantification (right) of wound healing assays for Hep3B cells and Huh7 cells cultured within the indicated LX-2 CM (scale bar, 500 μm). **I,** CCK8 assay analyzing the cell proliferation of Hepa1-6 cells cultured within CM of primary HSCs isolated from control or *Mettl3* cKO mice for 48 hours. **J,** Representative images (left) and quantification (right) of crystal violet staining for colony-forming assays for Hepa1-6 cells cultured within primary HSC CM for 7 days (scale bar, 200 μm). Data in **B–D** and **F–J** are shown as mean ± SEM with the indicated significance (*, *P* < 0.05; **, *P* < 0.01; ***, *P* < 0.001; ###, *P* < 0.001). CM, conditioned medium; mHSC, mouse primary HSC.

Then, we tested the HCC-promoting effects of METTL3 deficiency in both mouse primary HSCs and the human HSC cell line LX-2 *in vitro*. We first created the METTL3-knockdown LX-2 cells using 2 independent short hairpin RNAs (shRNA) and collected CM to assess their effects on cell proliferation and migration in human hepatoma cell lines Hep3B and Huh7 (Fig. 3A and B; Supplementary Fig. S1A). As expected, CM from METTL3-knockdown cells significantly elevated the proliferation of both Hep3B and Huh7 cells detected by Cell Counting Kit 8 (CCK8; Fig. 3C and D). The direct mixed coculture experiment of GFP-transduced hepatoma cells and LX-2 cells also showed that the number of both Hep3B and Huh7 cells increased by coculturing with METTL3-knockdown LX-2 cells (Fig. 3E and F). LX-2 cells pretreated with the METTL3 inhibitor STM2457 (19) also showed an increased number of GFP^+^ hepatoma cells (Supplementary Fig. S1B and S1C), confirming that knocking down METTL3 or inhibiting its activity in HSCs promoted the proliferation of HCC cells. Similarly, crystal violet staining displayed more hepatoma cell clones in the METTL3-knockdown LX-2 CM–treated group (Fig. 3G), suggesting that METTL3 depletion in HSCs promoted the colony formation of HCC cells. Furthermore, METTL3 deficiency also promoted the wound-healing rate of both Hep3B and Huh7 cells (Fig. 3H). Next, we validated the HCC-promoting effects of METTL3 deficiency in HSCs with mouse primary HSCs (Supplementary Fig. S1A). Consistent with the results in human LX-2 cells, the CM of primary HSCs from *Mettl3* cKO mice also increased the proliferation and colony formation of mouse hepatoma cells Hepa1-6 compared with that from control mice (Fig. 3I and J). These results confirmed that METTL3 deficiency in HSCs promoted HCC progression, at least in part via a paracrine mechanism.

### BMP10 was a direct target of METTL3-mediated m^6^A modification in HSCs

**Figure 4.**
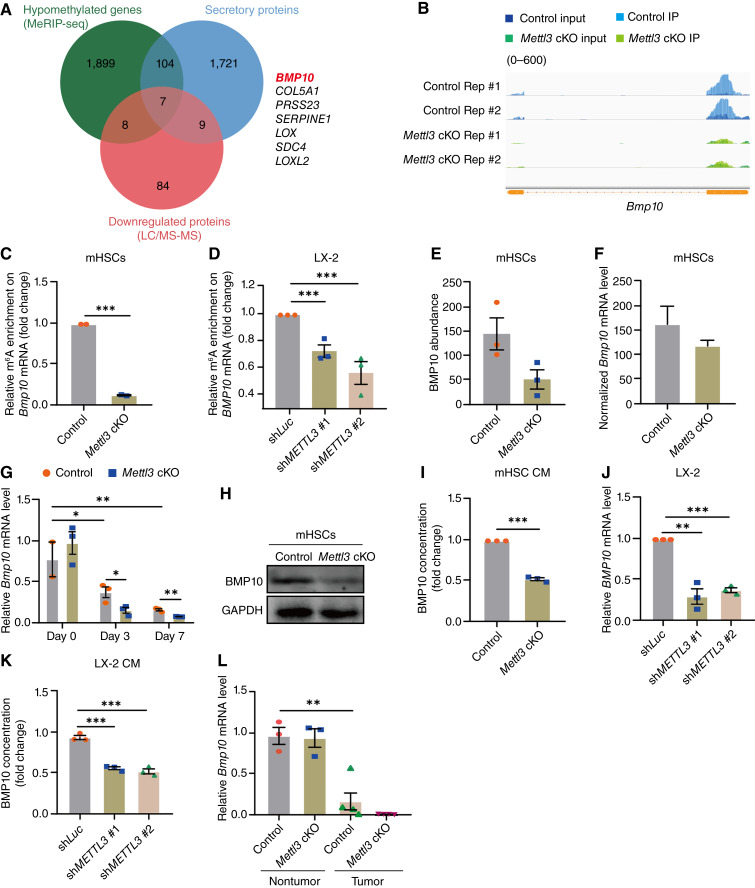
BMP10 was a direct target of METTL3-mediated m^6^A modification in HSCs. **A,** Venn diagram showing the overlapping genes among m^6^A hypomethylated genes (identified by MeRIP-seq) in primary HSCs isolated from *Mettl3* cKO mice activated *in vitro* compared with those from WT controls, genes encoding human secreted proteins (gene list was retrieved from https://www.proteinatlas.org/), and downregulated proteins in *Mettl3* cKO HSCs activated *in vitro* vs. WT control cells, analyzed by mass spectrometry (LC/MS-MS). **B,** MeRIP-seq read distribution profiles in the *Bmp10* locus of culture-activated primary HSCs isolated from control or *Mettl3* cKO mice (*n* = 2 for each group). **C,** Gene-specific m^6^A MeRIP-qPCR for relative m^6^A enrichment on *Bmp10* mRNA transcripts in culture-activated primary HSCs from control and *Mettl3* cKO mice (*n* = 2 for each group). **D,** MeRIP-qPCR for m^6^A enrichment on *BMP10* transcript of LX-2 cells transduced with sh*METTL3* or sh*Luc* lentivirus. **E,** LC/MS-MS analysis of BMP10 abundance in culture-activated primary HSCs isolated from control and *Mettl3* cKO mice (*n* = 3 for each group). **F,** RNA-seq revealing the relative mRNA level of *Bmp10* in culture-activated primary HSCs isolated from control and *Mettl3* cKO mice. **G,** RT-qPCR for *Bmp10* in primary HSCs from control and *Mettl3* cKO mice cultured *in vitro* for the indicated time (*n* = 2–3 for each group). **H,** Representative Western blots for BMP10 in culture-activated primary HSCs isolated from control and *Mettl3* cKO mice (*n* = 3 independent experiments). **I,** ELISA for the BMP10 concentration in CM of primary HSCs in indicated groups cultured *in vitro* for 72 hours (*n* = 3 independent experiments). **J,** RT-qPCR for *BMP10* in LX-2 cells transduced with sh*METTL3* or sh*Luc* lentivirus (*n* = 3 independent experiments). **K,** ELISA for the BMP10 concentration in CM of LX-2 cells transduced with sh*METTL3* or sh*Luc* lentivirus. **L,** RT-qPCR for *Bmp10* in tumor or adjacent nontumor liver tissues from orthotopic HCC models established in control and *Mettl3* cKO mice with liver fibrosis. Data in **C–G** and **I–L** are shown as mean ± SEM with the indicated significance (*, *P* < 0.05; **, *P* < 0.01; ***, *P* < 0.001). CM, conditioned medium; IP, immunoprecipitation; mHSC, mouse primary HSC.

To identify the underlying molecular mechanisms of the observed tumor-promoting effect of METTL3 deficiency in HSCs on HCC progression, we overlapped m^6^A hypomethylated genes (identified by MeRIP-seq) in primary HSCs isolated from *Mettl3* cKO mice and activated *in vitro*, compared with those from control HSCs, genes encoding human secreted proteins (gene list was retrieved from https://www.proteinatlas.org/), and differently expressed proteins in *Mettl3* cKO HSCs versus control HSCs activated *in vitro* analyzed by mass spectrometry, to identify key targets of METTL3/m^6^A mediating the cross-talk between HSCs and hepatoma cells. We found that 7 secretory proteins were hypomethylated and downregulated in *Mettl3* cKO HSCs (Fig. 4A; Supplementary Fig. S1D). Among them, BMP10, a reported HCC suppressor (20), caught our attention. BMP10 is a protein mainly secreted by fibroblasts and liver sinusoidal endothelial cells in liver tissues, belonging to the transforming growth factor β (TGFβ) superfamily, the expression of which is essential for heart development (21), cardiomyocyte differentiation (22), vascular development and maintenance (23, 24), and bone formation (25). In HCC, BMP10 negatively regulated HCC cell proliferation by suppressing STAT3 signaling (20).

Our MeRIP-seq data showed that m^6^A modification on *Bmp10* transcripts at the 3′ untranslated region was obviously decreased in *Mettl3* cKO HSCs, which was verified by MeRIP-qPCR (Fig. 4B–D). Mass spectrometry, RNA sequencing, RT-qPCR, and Western blotting showed that both protein and mRNA levels of BMP10 decreased in *Mettl3* cKO mouse HSCs compared with those from control mouse (Fig. 4E–H). Moreover, ELISA showed that the BMP10 concentration in the CM from *Mettl3* cKO HSCs also declined compared with control HSCs (Fig. 4I). Similarly, knocking down METTL3 in LX-2 cells decreased BMP10 expression and secretion (Fig. 4J and K). These data demonstrated that BMP10 was a direct target of METTL3/m^6^A modification in HSCs.

We also examined BMP10 expression in HCC tumors and nontumor tissues. Data from The Cancer Genome Atlas revealed a significant decrease in BMP10 expression in human HCC tissues (Supplementary Fig. S1E and S1F). Similarly, BMP10 levels were lower in tumor tissues compared with adjacent nontumor tissues in the mouse orthotopic HCC model with liver fibrosis (Fig. 4L). Interestingly, BMP10 showed a further decrease in tumor tissues in *Mettl3* cKO mice compared with control mice (Fig. 4L). Furthermore, by reclustering and reanalyzing the scRNA-seq data in GSE212046, we found that BMP10 was primarily expressed in HSCs and its expression level in HSCs from tumor tissues was much lower than that from nontumor tissues (Supplementary Fig. S1G and S1H). These findings suggested that the tumor-promoting effects of METTL3 depletion in HSCs may be associated with the concurrent decrease in BMP10.

### BMP10 mediated the tumor-promoting function of METTL3-deficient HSCs through SMAD1/5/8 phosphorylation

**Figure 5.**
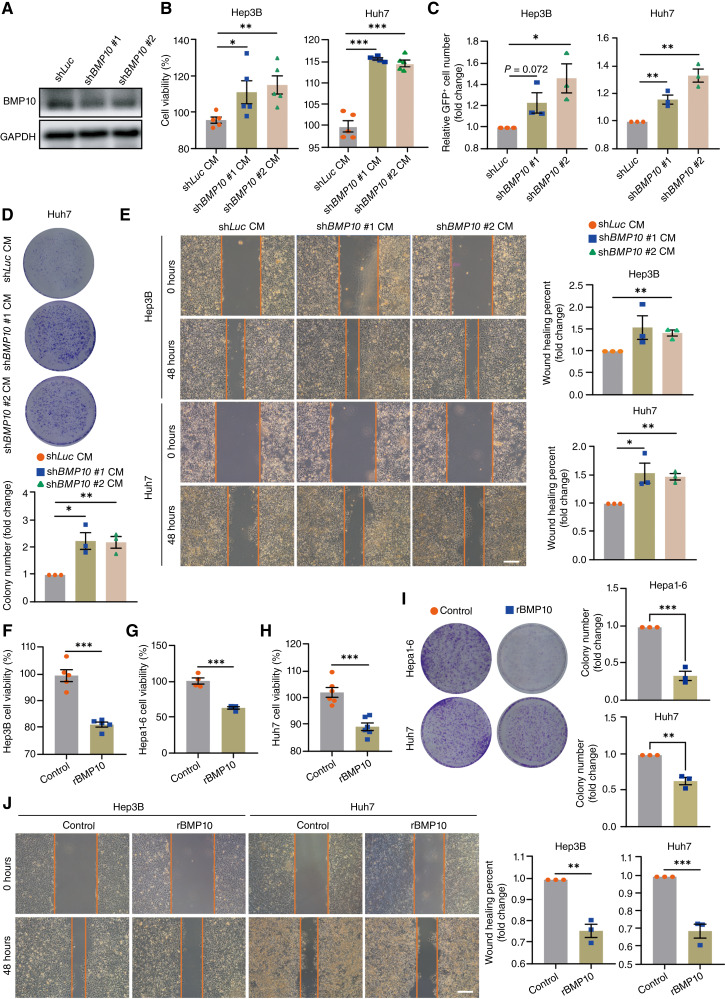
BMP10 deficiency in HSCs mirrors the tumor-boosting effects of METTL3 deficiency, whereas recombinant BMP10 has the opposite effect. **A,** Representative Western blots for BMP10 in LX-2 transduced with sh*Luc* or 2 independent shRNAs targeting *BMP10* (sh*BMP10* #1 and sh*BMP10* #2). **B,** CCK8 assay analyzing the cell proliferation of Hep3B cells (left) and Huh7 cells (right) cultured within CM from indicated LX-2 cells for 48 hours. **C,** Hepatoma cells were mixed cocultured with sh*BMP10* LX-2 or sh*Luc* LX-2 cells for 48 hours. The number of Hep3B cells (left) and Huh7 cells (right) was quantified by GFP signals using a flow cytometer. **D,** Representative images (up) and quantification (down) of crystal violet staining for colony-forming assays of Huh7 cells cultured within the indicated LX-2 CM for 7 days. **E,** Representative images (left) and quantification (right) of wound healing assays for Hep3B cells or Huh7 cells cultured within the indicated LX-2 CM for 48 hours. **F–H,** CCK8 assay analyzing the cell proliferation of Hep3B cells (**F**), Hepa1-6 cells (**G**), and Huh7 cells (**H**) cultured with or without human rBMP10 protein for 48 hours. **I,** Representative images (left) and quantification (right) of crystal violet staining for colony-forming assays of Hepa1-6 cells or Huh7 cells cultured with or without rBMP10 for 7 days. **J,** Representative images (left) and quantification (right) of wound healing assays for Hep3B cells or Huh7 cells cultured with or without rBMP10 for 48 hours (scale bar, 500 μm). Data in **B–J** are shown as mean ± SEM with the indicated significance (*, *P* < 0.05; **, *P* < 0.01; ***, *P* < 0.001).

**Figure 6.**
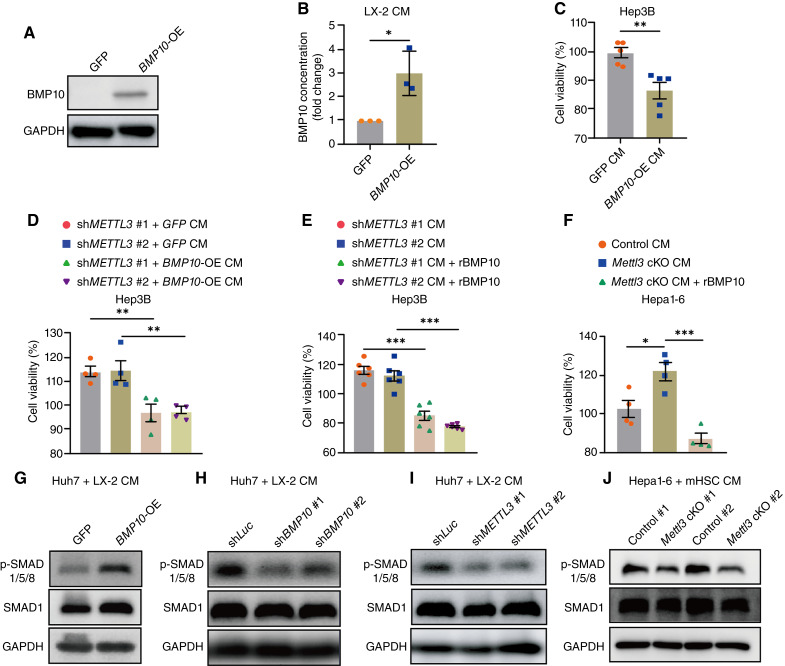
BMP10 mediates the tumor-promoting function of METTL3-deficient HSCs through SMAD1/5/8 phosphorylation. **A,** Representative Western blots for BMP10 in LX-2 transduced with GFP or *BMP10* overexpressing (*BMP10*-OE) lentivirus. **B,** ELISA for BMP10 concentration in the supernatant of LX-2 with GFP or *BMP10*-OE lentivirus treatment. **C,** CCK8 analysis for cell proliferation of Hep3B cells cultured within CM from indicated LX-2 cells for 48 hours. **D,** CCK8 analysis for cell proliferation of Hep3B cells cultured within CM from *METTL3-*knockdown LX-2 cells transduced with GFP or *BMP10* overexpressing lentivirus for 48 hours. **E,** CCK8 analysis for cell proliferation of Hep3B cells cultured within CM from *METTL3-*knockdown LX-2 cells with or without human rBMP10 protein for 48 hours. **F,** CCK8 analysis for cell proliferation of Hepa1-6 cells cultured within CM of primary HSCs isolated from control or *Mettl3* cKO mice with or without rBMP10 for 48 hours. **G,** Representative Western blots for phosphorylated-SMAD1/5/8 (p-SMAD1/5/8) and total SMAD1 in Huh7 cells cultured within CM from LX-2 cells transduced with GFP or *BMP10* overexpression lentivirus for 48 hours. **H,** Representative Western blots for p-SMAD1/5/8 and total SMAD1 in Huh7 cells cultured within CM from LX-2 cells transduced with sh*Luc* or sh*BMP10* lentivirus for 48 hours. **I,** Representative Western blots for p-SMAD1/5/8 and total SMAD1 in Huh7 cells cultured within CM from LX-2 cells transduced with sh*Luc* or sh*METTL3* lentivirus for 48 hours. **J,** Representative Western blots for p-SMAD1/5/8 and total SMAD1 in Hepa1-6 cells cultured within CM from primary HSCs isolated from control or *Mettl3* cKO mice for 48 hours. Data in **B–F** are presented as mean ± SEM with the indicated significance (*, *P* < 0.05; **, *P* < 0.01; ***, *P* < 0.001). mHSC, mouse primary HSCs.

To further confirm that downregulation of BMP10 directly mediates the tumor-promoting effects of METTL3/m^6^A-deficient HSCs, we constructed BMP10-knockdown LX-2 cells using 2 independent shRNAs (Fig. 5A; Supplementary Fig. S1I). Then, we treated hepatoma cell lines Hep3B and Huh7 with CM from LX-2 cells, with or without BMP10 knockdown. Similar to knocking down METTL3, BMP10 depletion in LX-2 resulted in a significant enhancement in the proliferation of Hep3B and Huh7 cells examined by CCK8 assay (Fig. 5B). Consistent results were observed in the mixed coculture experiments (Fig. 5C). Moreover, CM from LX-2 cells with BMP10 knockdown also enhanced colony formation and wound healing rate of hepatoma cells (Fig. 5D and E). In contrast, treatment with rBMP10 (10 ng/mL) significantly inhibited proliferation, colony formation, and wound-healing rate across different HCC cell lines (Fig. 5F–J). The supernatant from LX-2 cells overexpressing BMP10 via lentivirus transduction also inhibited the proliferation of Hep3B cells (Fig. 6A–C). These data suggested that depleting BMP10 in HSCs phenocopies the tumor-promoting effect of METTL3/m^6^A intervention in HSCs on hepatoma cells, whereas increasing BMP10 levels had the opposite effect.

To further confirm that downregulation of BMP10 mediated the tumor-promoting effect of METTL3/m^6^A deficiency in HSCs, we overexpressed BMP10 in METTL3-knockdown LX-2 cells to observe whether BMP10 overexpression could counteract the promoting effect of METTL3-knockdown HSCs on hepatoma cell proliferation. CM administration showed that BMP10 overexpression retrieved the proliferation-promoting effect induced by METTL3 knockdown (Fig. 6D). Moreover, rBMP10 (10 ng/mL) also abolished the proliferation-promoting function of METTL3-deficient HSCs on hepatoma cells (Fig. 6E and F). Our data demonstrated that BMP10, a secretory tumor suppressor, was a critical downstream target of METTL3-mediated m^6^A in HSCs, mediating the cross-talk between HSCs and hepatoma cells in HCC progression.

As one of the archetypal receptor–ligand–mediated signaling pathways, the canonical BMP10 signaling pathway is initiated when a dimeric ligand binds to its heterotetrameric receptor complex (type I and type II BMP receptors), leading to receptor phosphorylation. Upon phosphorylation, the receptors, in turn, recruit and phosphorylate SMAD1/5/8, which interacts with SMAD4 and subsequently translocates into the nucleus to further regulate gene transcription (26). To further confirm that BMP10 mediated the tumor-promoting function of METTL3-deficient HSCs, we measured SMAD1/5/8 phosphorylation levels in hepatoma cells treated with CM from HSCs with the indicated manipulation. As expected, CM from BMP10-overexpressing LX-2 cells induced SMAD1/5/8 phosphorylation (Fig. 6G), whereas CM from BMP10-knockdown LX-2 cells reduced SMAD1/5/8 phosphorylation in HCC cells (Fig. 6H). Interestingly, consistent with BMP10 inhibition, CM from both METTL3-knockdown LX-2 cells and Mettl3-KO primary HSCs decreased the SMAD1/5/8 phosphorylation level of hepatoma cells (Fig. 6I and J). These data further verified that the BMP10/SMAD1/5/8 pathway mediated the tumor-promoting function of METTL3-deficient HSCs.

## Discussion

Approximately 80% to 90% of HCC develops in a fibrotic or cirrhotic liver (27). The activation of HSCs is a central event and driver of liver fibrosis. In a healthy liver, HSCs are in a quiescent, low-proliferative state. In response to chemical toxins, alcohol, viruses, or other liver injury factors, HSCs are activated into myofibroblast-like cells and produce ECM to prevent further damage (28). However, with long-term injury stimulation, activated HSCs in a high-proliferative state persistently produce much more ECM, leading to chronic fibrosis and cirrhosis gradually, part of which eventually develops into HCC. It has been reported that activated HSCs are involved in HCC onset, growth, angiogenesis, metastasis, and drug resistance, suggesting that activated HSCs play a critical role in the HCC microenvironment.

Although previous studies have found that METTL3, the core component of the “writers” of m^6^A methylation, was significantly upregulated in hepatoma cells and that increased METTL3 promoted HCC development (29, 30), our study revealed an opposite phenotype and function of METTL3 in HSCs infiltrating the HCC microenvironment. In this study, we found that METTL3 decreased gradually as HSC activation increased. Our further exploration revealed that the number of activated HSCs in human HCC samples was increased and METTL3 expression of HSCs was downregulated. Functional analysis showed that METTL3 deficiency in HSCs facilitated HCC growth and progression *in vitro* and *in vivo*. We speculate that METTL3 exhibits distinct expression patterns in specific cell types and plays multiple roles in regulating the overall progression of HCC, suggesting that future METTL3-targeted cancer therapies will require cell-specific drug-delivery systems to achieve optimal therapeutic efficacy.

Furthermore, METTL3 in HSCs seems to play distinct roles at different stages of disease progression. Knockout of METTL3 in HSCs inhibits HSC activation and fibrosis progression (10) but paradoxically promotes tumor progression in the fibrotic liver in this study. We hypothesize that this discrepancy is primarily driven by 2 key factors: First, activated HSCs are not merely collagen-secreting fibrogenic cells; they also undergo significant reprogramming of their secretome to modulate the liver microenvironment (4). Second, distinct roles across disease stages: The impact of HSCs/fibrosis likely differs between tumor initiation and progression. It is plausible that although fibrosis promotes mutation accumulation during initiation, activated HSCs in the progression phase may exert complex, context-dependent effects on existing tumor masses that are independent of, or even opposing to, their fibrotic activity.

Unlike the hepatoma cells themselves, as a key component of the HCC TME, activated HSCs and their derived CAFs crosstalk with tumor cells and immune cells in the TME through paracrine signaling to remodel the TME and further facilitate tumor formation, metastasis, and chemoresistance (5, 31). In this work, we identified another m^6^A target gene, *BMP10*, in HSCs. Mechanistically, we found that the m^6^A methylation was less abundant in *Bmp10* transcripts in primary HSCs from *Mettl3*-cKO mice, accompanied by decreased BMP10 expression at both protein and mRNA levels. BMP signaling is essential for certain developmental processes and the homeostasis of various tissues and organs, including myocardium (21), skeletal (26), vascular (23), and bone (32). Currently, aberrant BMP expression has been observed in a variety of solid tumors, and BMP10 has been identified as a tumor suppressor in HCC (20), breast cancer (33), prostate cancer (34), and osteosarcoma (35). As a secretory protein, BMP10 is shown to be principally secreted by HSCs in the liver (36, 37). In the present study, we found that blocking BMP10 expression in HSCs accelerated the proliferation and migration of HCC cells, whereas elevating BMP10 production from HSCs or rBMP10 protein did the opposite, phenocopying METTL3 intervention in HSCs. Moreover, both rBMP10 protein and BMP10 overexpression reversed the tumor-promoting effect of METTL3-deficient HSCs, consolidating that BMP10 is a core target of METTL3/m^6^A in HSCs, mediating the cross-talk between HSCs and HCCs.

Interestingly, although both our study and previous reports have established that BMP10 is primarily secreted by HSCs and possesses tumor-suppressive function (20, 36), other studies have shown that HSC-specific double-KO of BMP9 and BMP10 disrupts liver homeostasis and leads to spontaneous liver fibrosis (37). Therefore, the specific role of BMP10 in liver fibrosis and its subsequent impact on HCC progression via the fibrotic microenvironment warrants further investigation. It is generally recognized that TGFβ signaling activates SMAD2/3; meanwhile, receptors activated by BMP ligands mainly phosphorylate SMAD1/5/8. It is reported that BMP–SMAD1/5/8 signaling reduces the proliferative and migratory abilities of endothelial cells (38, 39). Hence, we hypothesized that BMP10 secreted by HSCs might regulate HCC progression by phosphorylating SMAD1/5/8. As expected, either METTL3 or BMP10 knockdown in LX-2 cells significantly reduced SMAD1/5/8 phosphorylation in hepatoma cells, whereas BMP10 overexpression had the opposite effect. Although our study identifies the BMP10–SMAD1/5/8 axis as a critical mediator of HSC-specific METTL3-driven HCC progression, the complex landscape of m^6^A methylation suggests that this is likely not the sole mechanism. Given that m^6^A modification orchestrates the expression of thousands of genes (40) and considering our previous identification of LATS2 as another METTL3/m^6^A target in HSCs (10), it is plausible that other downstream effectors cooperate with BMP10 to regulate tumor progression. Furthermore, we cannot exclude the possibility that METTL3/m^6^A-mediated modulation of BMP10 also influences cross-talk between HSCs and other resident or circulating liver cells, thereby contributing to HCC initiation.

In summary, our study found that METTL3 expression in HSCs infiltrating HCC tissues was markedly downregulated. Blocking METTL3 in HSCs showed a tumor-promoting effect on HCC by reducing the BMP10 secretion and subsequent phosphorylation of SMAD1/5/8 in HCC cells (Fig. 6K). Our study revealed a novel cross-talk between HSCs and hepatoma cells mediated by the METTL3–BMP10–SMAD1/5/8 axis, providing a potential strategy for HCC treatment.

## Supplementary Material

Supplementary Figure 1 Supplementary figure and legend

Supplementary Methods Supplementary materials and methods

Supplementary Table 1 Supplementary Table 1: Antibodies.

Supplementary Table 2 Supplementary Table 2:Primers

Supplementary Table 3 Supplementary Table 3:Reagents

## Data Availability

The data generated in this study are available upon request to the corresponding author.
