# Supplementary material for: Hepatic Stellate Cell–Specific METTL3 Deficiency Promotes Hepatocellular Carcinoma Progression via BMP10–SMAD1/5/8 Signaling
Source: Cancer Res Commun. 2026 May 13;6(5):1109–22. doi: 10.1158/2767-9764.CRC-25-0761 (PMC13168861; doi:10.1158/2767-9764.CRC-25-0761)
Supplement: Supplementary Figure 1 — Supplementary figure and legend [file crc-25-0761_supplementary_figure_1_suppsf1.pdf]

Supplementary figure

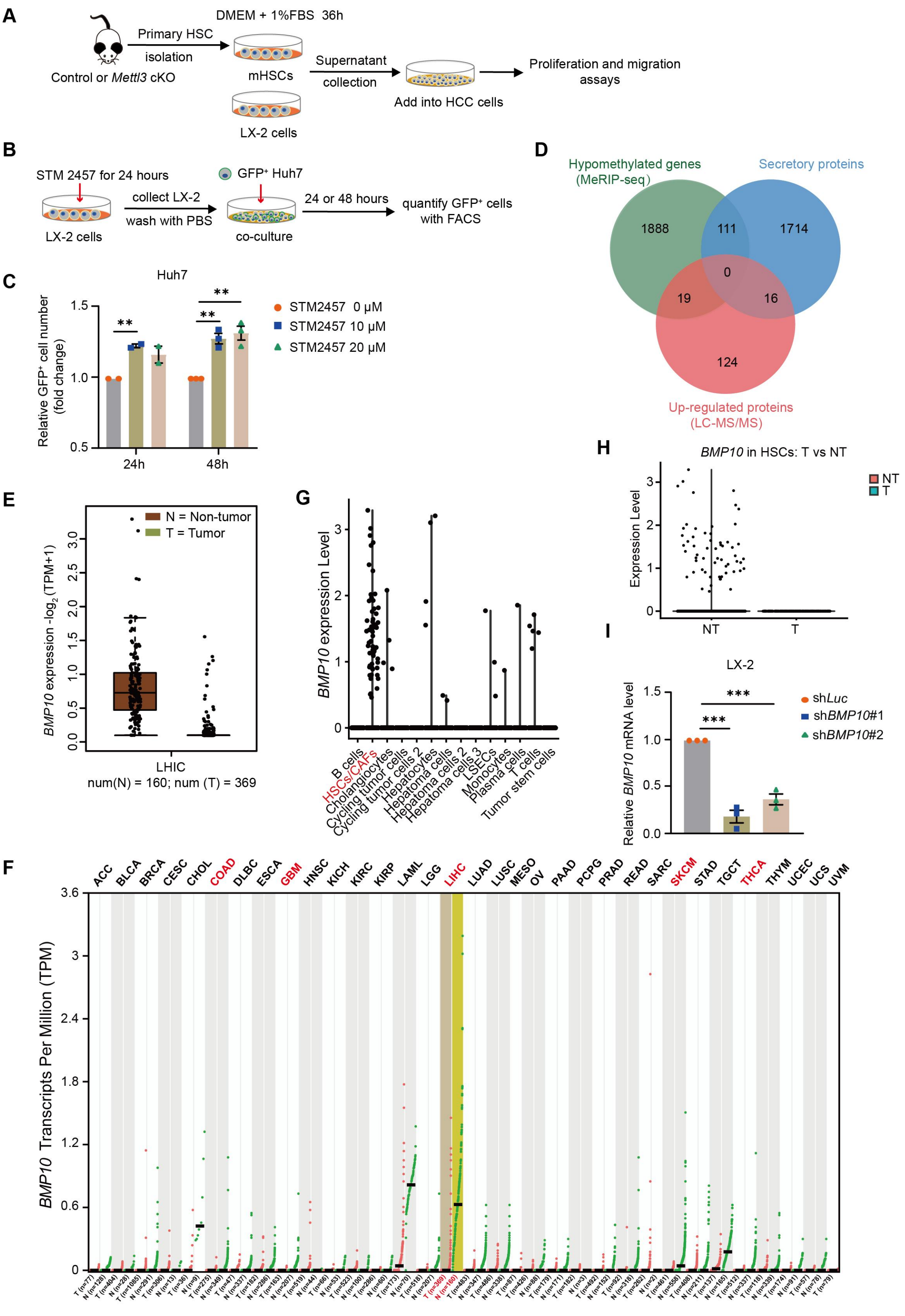

Figure. S1

(A) Schematic diagram of indirect co-culture of HSCs and hepatoma cells. Conditioned medium was collected from primary HSCs or LX-2 cells incubated in medium containing 1% FBS for 36 hours. (B & C) LX-2 cells were pretreated with the indicated concentration of METTL3 inhibitor STM2457 for 24 hours, then washed thoroughly with PBS to remove residual inhibitors, and mixed co-cultured with GFP-transduced Huh7 cells for 24 or 48 hours. (B) Schematic diagram of experimental design. (C) FACS quantification of the GFP^+ Huh7 cells in the indicated groups. (D) Venn diagram demonstrates the overlapping genes among m^6A hypomethylated genes (identified by MeRIP-seq) in primary HSCs isolated from *Mettl3* cKO mouse activated *in vitro* compared to those from wild-type controls, genes encoding human secreted proteins (gene list was retrieved from https://www.proteinatlas.org/), and up-regulated proteins in *Mettl3* cKO HSCs activated *in vitro* versus wild-type control cells analyzed by mass spectrometry. (E) Expression profile of BMP10 in tumor and non-tumor tissues across various tumor types in the TCGA database. The non-tumor data included both para-tumor data from the TCGA database and healthy control data from the GTEX database. Analysis was conducted using GEPIA (http://gepia.cancer-pku.cn/). (F) Expression profile of BMP10 in tumor and non-tumor tissues of HCC in the TCGA database. The non-tumor data included both para-tumor data from the TCGA database and healthy control data from the GTEX database. (G) *BMP10* expression in various cell types in hepatocellular carcinoma (scRNA sequencing data of GSE212046). (H) *BMP10* expression level of HSCs in tumor (T) or non-tumor (NT) group (scRNA sequencing data of GSE212046). (I) RT-qPCR for *BMP10* in LX-2 transduced with sh*Luc* or 2 independent shRNAs targeting *BMP10*. Data in (C) and (I) was presented as mean ± SEM with the indicated significance ($^{**}p < 0.01$, $^{***}p < 0.001$). Abbreviations: HSC, hepatic stellate cells; FBS, fetal bovine serum.
