# Supplementary material for: Hepatic Stellate Cell–Specific METTL3 Deficiency Promotes Hepatocellular Carcinoma Progression via BMP10–SMAD1/5/8 Signaling
Source: Cancer Res Commun. 2026 May 13;6(5):1109–22. doi: 10.1158/2767-9764.CRC-25-0761 (PMC13168861; doi:10.1158/2767-9764.CRC-25-0761)
Supplement: Supplementary Methods — Supplementary materials and methods [file crc-25-0761_supplementary_methods_suppsm.pdf]

Supplementary Materials and Methods

Liver biochemical measurement

Serum levels of alanine aminotransferase (ALT), aspartate aminotransferase (AST), alkaline phosphatase (ALP), and albumin (ALB) were measured using Hitachi 7020 automatic biochemical analyzer (Hitachi, Tokyo, Japan).

H&E and immunohistochemical staining

Liver tissues were fixed in 4% paraformaldehyde, embedded in paraffin, and sectioned into 5 μm slides for further hematoxylin and eosin (H&E) or immunohistochemical staining. For immunohistochemical staining, liver sections were routinely dewaxed, rehydrated, and antigen retrieved, then incubated with primary antibodies (α-SMA, 1:200, Abcam, ab5694, RRID: AB_2223021; AFP, 1:200, Abcam, ab290637) at 4°C overnight. Secondary antibodies against rabbit IgG (1:100, Cell Signaling Technology, 7074, RRID: AB_2099233) were subsequently incubated at room temperature for 1 h. The image was viewed and captured by an optical microscope (Nikon, Japan).

Sphere formation assay and crystal violet staining

The hepatoma cells Hep3B, Huh7, and Hepa1-6 were seeded in 12-well plates at a density of 1×10^4 cells per well and cultured overnight. Subsequently, the cells were incubated with the indicated conditioned medium (CM) from primary HSCs or LX-2 cells for one week. At scheduled time points, the cells were fixed with 4% paraformaldehyde for 10 minutes, stained with 0.1% crystal violet for 10 minutes, and

the spheres were observed and scanned.

Enzyme-linked immunosorbent assay (ELISA)

The BMP10 concentration in the culture supernatant of indicated primary HSCs and LX-2 cells was quantitated by Human/Mouse BMP-10 DuoSet ELISA kit (R&D, DY2926-05)) according to the manufacturer's instructions.

Cell proliferation and migration assay

Cell proliferation analyses were performed by Cell Counting Kit-8 (CCK-8) (APExBIO, K1018). Wound-healing assays were used to assess the cell migratory ability. Hepatoma cells were incubated with CM from primary HSCs or LX-2 cells for the indicated time. For the CCK-8 assay, the CCK-8 solution was added directly into the 96-well plate at a 1:10 (v/v) ratio and reacted in a routine culture environment for 1 hour. The absorbance at 450nm was tested by Microplate Reader (TECON, Switzerland), and cell viability was calculated.

Cell culture

293T cells (RRID: CVCL_0063) were purchased from the American Type Culture Collection (ATCC) and maintained in DMEM-high glucose medium supplemented with 10% FBS and incubated at 37 °C in a humidified atmosphere of 5% CO_2. Cells were continuously monitored for mycoplasma contamination to ensure they remained free of contamination.

Plasmid construction and virus transduction

shRNA targeting human *METTL3* (sh*METTL3*), *BMP10* (sh*BMP10*), or firefly luciferase (sh*Luc*) were cloned into pLKO.1 lentiviral vector (Addgene, 10878). Human METTL3 or BMP10 over-expression plasmids were established by PKD-EF1 lentiviral vector. PKD-EF1 vector expressing EGFP was used as a control. Lentivirus transfections were performed using PEI (Polysciences, 23966). Briefly, 293T cells were seeded into 6 cm dished one day before, and transfection was conducted when cells reached 90-95% confluence. For each well, 336 μL of pre-warmed OptiMEM (Invitrogen) was mixed with 8 μg of plasmids (target plasmid : psPAX2 : pMD2.G = 3 : 2 : 1) and 36 μL of PEI (1 μg/mL). After reacting for 10-12 minutes at room temperature, the mixture was added dropwise into the culture medium, and the medium was changed 12 hours later. After incubation for 48 hours, the supernatants were collected and filtrated with a 0.45 μm filter. Target cells were infected with the lentivirus-containing supernatants with 1 μg/mL polybrene (Sigma, H9268) for 6-8 hours. Cells were then selected with 1 μg/mL puromycin (Thermo Scientific, A1113803). Primers used for plasmid construction were listed in *Supplementary Table 2*.

Reference

1. Li Y, Kang X, Zhou Z, Pan L, Chen H, Liang X, et al. The m(6)A methyltransferase Mettl3 deficiency attenuates hepatic stellate cell activation and liver fibrosis. Mol Ther. 2022;30(12):3714-28.
