# Supplementary material for: Hepatic Stellate Cell–Specific METTL3 Deficiency Promotes Hepatocellular Carcinoma Progression via BMP10–SMAD1/5/8 Signaling
Source: Cancer Res Commun. 2026 May 13;6(5):1109–22. doi: 10.1158/2767-9764.CRC-25-0761 (PMC13168861; doi:10.1158/2767-9764.CRC-25-0761)
Supplement: Supplementary Table 1 — Antibodies. [file crc-25-0761_supplementary_table_1_suppst1.pdf]

Supplementary Table 1. Antibodies

Antibodies	Source	Identifier
m^6A (N6-methyladenosine) antibody (RRID: AB_2279214)	Synaptic Systems	Cat# 202003
mouse IgG (RRID: AB_2909433)	Beyotime	Cat# A7028
Anti rabbit IgG HRP-linked antibody (RRID: AB_2099233)	Cell Signaling Technology	Cat# 7074
anti-BMP10 antibody (RRID: AB_2065650)	R&D systems	Cat# MAB2926-100
Anti p-SMAD1/5/8 antibody (RRID: AB_2493181)	Cell Signaling Technology	Cat# 13820
Anti GAPDH antibody (RRID: AB_561053)	Cell Signaling Technology	Cat# 2118
Anti SMAD1 antibody (RRID: AB_10858882)	Cell Signaling Technology	Cat# 6944T
