# Supplementary material for: Hepatic Stellate Cell–Specific METTL3 Deficiency Promotes Hepatocellular Carcinoma Progression via BMP10–SMAD1/5/8 Signaling
Source: Cancer Res Commun. 2026 May 13;6(5):1109–22. doi: 10.1158/2767-9764.CRC-25-0761 (PMC13168861; doi:10.1158/2767-9764.CRC-25-0761)
Supplement: Supplementary Table 2 — Primers [file crc-25-0761_supplementary_table_2_suppst2.pdf]

Supplementary Table 2. Primers

1. Primers for genotyping

Name	Sequences
Mettl3-F1	5'-AGAGGAGGAGAAGGTGGCAGAG-3'
Mettl3-R1	5'-CCTTTCATTCACATGGCAGCAC-3'
Mettl3-R2	5'-AGGCCTATAATCCTAGCACTG-3'
Lrat-Cre-F	5'-TGAGCCAAGCACTTTGGCTTC-3'
Lrat-Cre-R	5'-TCACATCCTCAGGTTCAGCAGG-3'

2. Primers for RT-qPCR (mouse)

Name	Sequences
mouse *Gapdh*-RT-F	5'-CATGGCCTTCCGTGTTCCT-3'
mouse *Gapdh*-RT-R	5'-GCCTGCTTCACCACCTTCT-3'
mouse *Bmp10*-RT-F	5'-CACCAGAGTACATGCTGGAGCT-3'
mouse *Bmp10*-RT-R	5'-GGATAGACACATTGAAGAGGAGAG-3'

3. Primers for RT-qPCR (human)

Name	Sequences
human *GAPDH*-RT-F	5'-AATGAAGGGGTCATTGATGG-3'
human *GAPDH*-RT-R	5'-AAGGTGAAGGTCGGAGTCAA-3'
human *BMP10*-RT-F	5'-TGCAACAGATCGGACCTCCATG-3'
human *BMP10*-RT-R	5'-GGAATGGACACATTGAAGAGGAG-3'
human *METTL3*-RT-F	5'-GAAGCAGCTGGACTCTCTGC-3'
human *METTL3*-RT-R	5'-ACGGAAGGTTGGAGACAATG-5'

4. Primers for m^{6}A-RIP-qPCR

Name	Sequences
mouse *Bmp10*-m^{6}A-RT-F	5'-ACCCATCAGCTGGAGATCCAC-3'
mouse *Bmp10*-m^{6}A-RT-R	5'-AACCAGCAAAGGGTCATGCT-3'
human *BMP10*-m^{6}A-RT-F	5'-GGACAACTTGGGCCTGGATA-3'

human *BMP10*-m^{6}A-RT-R	5'-GATTCGGGCAGTGGAGTCAT-3'

5. Primers for TRC lentiviral vectors of shRNA and overexpression construction

**Name**	**Sequences**
sh*METTL3*-1-F	5'-CCGGCGTCAGTATCTTGGGCAAGTTCTCGAGAACTTGCCCAAGATACTGACGTTTTTG-3'
sh*METTL3*-1-R	5'-AATTCAAAAA CGTCAGTATCTTGGGCAAGTTCTCGAGAACTTGCCCAAGATACTGACG-3'
sh*METTL3*-2-F	5'-CCGGGCCAAGGAACAATCCATTGTTCTCGAGAACAATGGATTGTTCCTTGGCTTTTTG-3'
sh*METTL3*-2-R	5'-AATTCAAAAA GCCAAGGAACAATCCATTGTTCTCGAGAACAATGGATTGTTCCTTGGC-3'
sh*BMP10*-1-F	5'-CCGGCAGAGCATGAAGGATGAGTTTCTCGAGAAACTCATCCTTCATGCTCTGTTTTTG-3'
sh*BMP10*-1-R	5'-AATTCAAAAACAGAGCATGAAGGATGAGTTTCTCGAGAAACTCATCCTTCATGCTCTG-3'
sh*BMP10*-2-F	5'-CCGGCGGCTAGAAATAGATACCAGTCTCGAGACTGGTATCTATTTCTAGCCGTTTTTG-3'
sh*BMP10*-2-R	5'-AATTCAAAAACGGCTAGAAATAGATACCAGTCTCGAGACTGGTATCTATTTCTAGCCG-3'
sh*BMP10*-3-F	5'-CCGGCCCATCTCCATCCTCTATTTACTCGAGTAAATAGAGGATGGAGATGGGTTTTTG -3'
sh*BMP10*-3-R	5'-AATTCAAAAACCCATCTCCATCCTCTATTTACTCGAGTAAATAGAGGATGGAGATGGG -3'
BMP10 overexpression-F	5'- AAACTACGGGATCCATGGGCTCTCTGGTCC-3'
BMP10 overexpression-F	5'- CGAATGTGGCTGTAGAGAATTCGACTACAAGGAC-3'
