# Supplementary material for: Hepatic Stellate Cell–Specific METTL3 Deficiency Promotes Hepatocellular Carcinoma Progression via BMP10–SMAD1/5/8 Signaling
Source: Cancer Res Commun. 2026 May 13;6(5):1109–22. doi: 10.1158/2767-9764.CRC-25-0761 (PMC13168861; doi:10.1158/2767-9764.CRC-25-0761)
Supplement: Supplementary Table 3 — Reagents [file crc-25-0761_supplementary_table_3_suppst3.pdf]

Supplementary Table 3. Reagents

Reagents	Source	Identifier
ChamQ Universal SYBR qPCR Master Mix	Vazyme	Q711-03
ClonExpress II One-Step Cloning kit	Vazyme	C112
Pronase	Sigma-Aldrich	11459643001
Nycodenz	Accurate Chemical	1002424
Collagenase IV	Sigma-Aldrich	C5138
Recombinant Human BMP-10 Protein	R&D systems	2926-BP-025
Complete Protease Inhibitor	Roche	04693132001
Direct Mouse Genotyping kit	ApexBio Technology	K1025
DMEM-high glucose medium	Thermo Scientific	C11995500BT
EDTA antigen retrieval buffer	ZSGB-BIO	ZLI-9072
Fetal bovine serum	PAN	P30-3302
GenElute™ mRNA Miniprep Kit	Sigma-Aldrich	MRN10
Hematoxylin	Baso	BA4041
Immonilon ECL Ultra Western HRP Substrate	Millipore	WBULS0500
Polyethylenimine (PEI)	Polysciences	23966
Penicillin/streptomycin	KeyGEN Biotech	KGY0023
Phosphatase inhibitor	Roche	04906831001
Polybrene	Sigma-Aldrich	H9268
HiScript III One Step RT-PCR Kit	Vazyme	R323-01
Protein A beads	Thermo Scientific	100-02D
Protein G beads	Thermo Scientific	100-04D
Puromycin	Thermo Scientific	A1113803
RNasin® Ribonuclease Inhibitor-plus	Promega	N2611

Stbl3 *E. coli*	TransGen Biotech	CD521-01
Triton^{TM} X-100	Sigma-Aldrich	T8787
TRIzol reagent	Invitrogen	15596026
Tween-20	Sigma-Aldrich	P1379
Olive oil	MACKLIN	O815211
Carbon tetrachloride (CCl_4)	Sigma-Aldrich	289116
pLKO.1 Vector (RRID:Addgene_10878)	Addgene	10878
